# Acneiform Eruptions Possibly Triggered by Clarithromycin During Sirolimus Treatment

**DOI:** 10.7759/cureus.61084

**Published:** 2024-05-25

**Authors:** Koki Kataoka, Saeko Nakajima, Takashi Nomura, Kenji Kabashima

**Affiliations:** 1 Dermatology, Kyoto University Graduate School of Medicine, Kyoto, JPN; 2 Drug Discovery for Inflammatory Skin Diseases, Kyoto University Graduate School of Medicine, Kyoto, JPN; 3 Drug Development for Intractable Diseases, Kyoto University Graduate School of Medicine, Kyoto, JPN; 4 A*STAR Skin Research Labs (A*SRL), Agency for Science, Technology and Research (A*STAR), Singapore, SGP; 5 Singapore Immunology Network (SIgN), Agency for Science, Technology and Research (A*STAR), Singapore, SGP

**Keywords:** egfr, mtor, cyp3a, drug-drug interactions, clarithromycin, sirolimus, acneiform eruption

## Abstract

Acneiform eruption is the recognized dermatological side effect of sirolimus, an inhibitor of the mammalian target of rapamycin, although the pathophysiological mechanisms and dose dependency of this side effect remain unclear. This case report describes a case of a 40-year-old Japanese woman treated with systemic sirolimus who developed acneiform eruptions following the administration of clarithromycin. The acneiform eruption resolved after discontinuation of sirolimus and relapsed with the resumption. Since sirolimus and clarithromycin have a potential drug-drug interaction mediated by cytochrome P450 3A (CYP3A), this case suggests that the acneiform eruption developed in association with elevated blood levels of sirolimus. We conclude that clinicians should be aware of the possibility of developing acneiform eruption during sirolimus treatment, especially when administered with medications that inhibit CYP3A.

## Introduction

Sirolimus is one of the mammalian targets of rapamycin (mTOR) inhibitors, which forms an immunosuppressive complex with the intracellular protein FK506-binding protein family (FKBP) 12, inhibiting the activation of the kinase TOR. This inhibition blocks cell cycle progression, leading to immunosuppressive and anti-tumor effects [1]. Sirolimus is used for treating lymphangioleiomyomatosis and as an immunosuppressant after organ transplantation. Acneiform eruption is well known as a dermatological adverse effect associated with sirolimus treatment. Sirolimus-induced acneiform eruptions occur in 13-46% of users, with a male predominance. These eruptions typically appear on the face, trunk, and extremities, especially in seborrheic areas, and are characterized by inflammatory lesions with few comedones or cysts and rarely nodules [2]. However, the pathophysiological mechanisms and the potential dose-dependency of this condition have not been elucidated. Clarithromycin is a macrolide antibiotic that inhibits RNA-dependent protein synthesis by reversibly binding to the 50S ribosomal subunit of susceptible bacteria [3]. Clarithromycin inhibits cytochrome P450 3A (CYP3A)-mediated drug clearance by forming a metabolic intermediate complex (MIC) with CYP3A, thus contributing to increased blood levels of sirolimus [4]. Here, we report a case of acneiform eruptions potentially triggered by clarithromycin during sirolimus treatment.

## Case presentation

**Figure 1:**
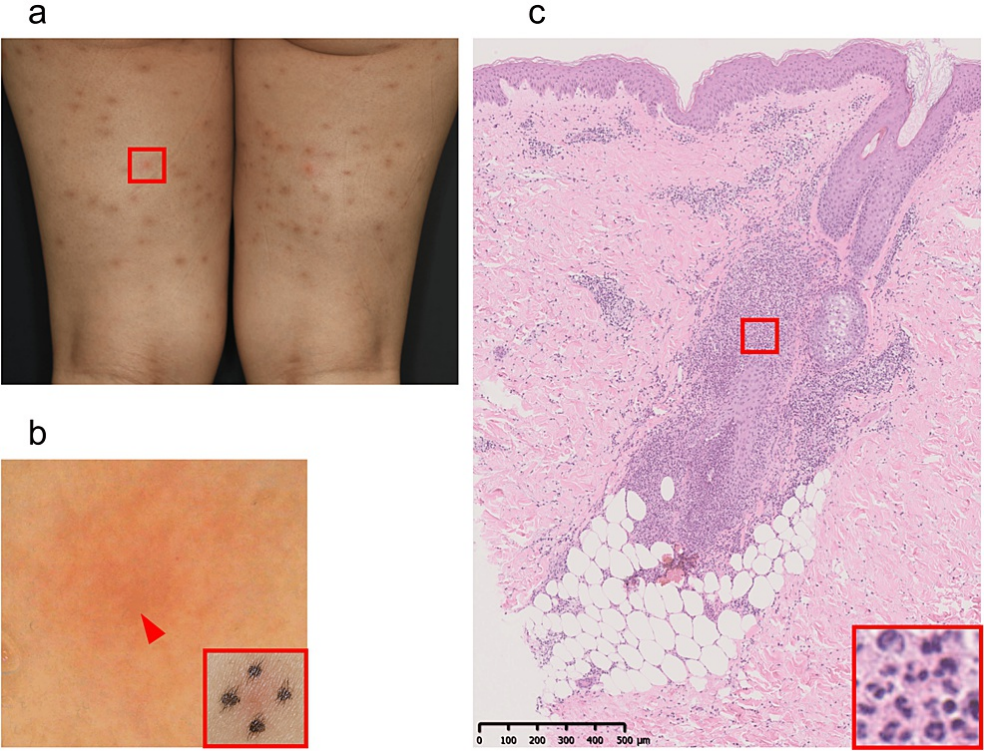
Clinical and histological features of acneiform eruption Multiple erythematous papules 2–5 mm in size with a mixture of old and new lesions on the posterior aspect of the thighs (a). The red frame is enlarged (a-bottom, right). Dermoscopy revealed hairs, indicated by the red arrowhead, in the center of the rashes, indicating that the rash had follicular consistency (b). A biopsy was taken from the same site. A dense perifollicular and perivascular inflammatory cell infiltrate was observed from the shallow to deep dermis (hematoxylin and eosin (c). The enlarged red box shows that inflammatory cells were predominantly neutrophils, with a few plasma cells and lymphocytes scattered (c-bottom, right). Neutrophil infiltration of the follicular and sebaceous epithelium was also spread.

A 40-year-old Japanese woman was referred to our clinic with recurrent acneiform eruptions on her body. She had started oral sirolimus 2 mg daily for lymphangioleiomyomatosis four months before her first presentation. Three months after initiating sirolimus, clarithromycin 400 mg/day was administered for pharyngeal bacterial infection. Four days after initiating clarithromycin, acne-like eruption developed over her lower extremities and trunk. Clarithromycin was discontinued the same day, and sirolimus was discontinued 15 days later. After discontinuation of sirolimus, the rash gradually disappeared. Sirolimus was resumed seven days before the first presentation, and the eruption recurred five days after re-initiating sirolimus. Physical exam revealed sporadic folliculocentric red papules 2-5mm in diameter on her proximal lower extremities with pruritus (Figures 1a, 1b). Skin biopsy showed folliculitis with dense neutrophil infiltration without any bacterial or fungal bodies (Figure 1c).

The blood tests on her first presentation are shown in Table 1 with no significant abnormalities.

**Table 1:** Blood tests on the initial presentation WBC: white blood cell; RBC: red blood cell; HGB: hemoglobin; HCT: hematocrit; MCV: mean corpuscular volume; MCH: mean corpuscular hemoglobin; MCHC: mean corpuscular hemoglobin concentration; PLT: platelet; PT-INR: prothrombin time-international normalized ratio; APTT: activated partial thromboplastin time; eGFR: estimated glomerular filtration rate; LFTs: liver function tests; ALT: alanine aminotransferase; AST: serum aspartate aminotransferase; GGT: gamma-glutamyl transferase; LDH: lactate dehydrogenase; CK: creatine kinase; CRP: C-reactive protein; NGSP: national glycohemoglobin standardization program

Test	Results/Units	Normal Range
Complete Blood Count		
WBC	5.93 x 10^3^/µL	2.80 - 9.00
Neutrophil	54.0 %	46.0 - 62.0
Lymphocyte	32.5 %	30.0 - 40.0
Monocyte	12.5 %	4.0 - 7.0
Eosinophil	0.7 %	3.0 - 5.0
Basophil	0.3 %	0.0 - 1.0
RBC	5.75 x 10^6^/µL	3.59 - 4.80
HGB	15.3 g/dL	10.7 - 14.5
HCT	44.9 %	32.2 - 43.0
MCV	78.1 fL	78.7 - 99.7
MCH	26.6 pg	26.1 - 33.6
MCHC	34.1 %	32.4 - 34.7
PLT	275 x 10^3^/µL	128 - 347
Coagulation Profile		
Prothrombin Time	12.5 seconds	10 - 13
Prothrombin Time Activity	93 %	70 - 120
PT-INR	1.04	0.8 - 1.2
APTT	28.9 seconds	24 - 39
Fibrinogen	258 mg/dL	200 - 400
Urea and Electrolytes		
Sodium	139 mEq/L	137 - 144
Potassium	4.2 mEq/L	3.6 - 4.8
Chloride	106 mEq/L	101 - 108
Creatinine	0.69 mg/dL	0.46 - 0.78
Uric Acid	4.4 mg/dL	2.6 - 5.6
Urea Nitrogen	15 mg/dL	8 - 22
eGFR	74.6 mL/min/1.73m^2^	>60
LFTs		
ALT	15 IU/L	7 - 27
AST	17 IU/L	12 - 30
GGT	11 IU/L	7 - 29
Alkaline Phosphatase	226 IU/L	115 - 359
LDH	180 IU/L	124 - 226
CK	127 IU/L	43 - 157
Total Bilirubin	0.5 mg/dL	0.3 - 1.3
Total Protein	7.0 g/dL	6.3 - 8.1
Albumin	4.1 g/dL	3.9 - 5.1
CRP	<0.1 mg/dL	<0.2
Glucose Random	117 mg/dL	65 - 105
Hemoglobin A1c (NGSP)	5.9 %	4.6 - 6.2

Because of the lack of evident pus, we were unable to perform a bacterial culture. A skin biopsy from the papules on her proximal lower extremities revealed folliculitis with dense neutrophil infiltration without any bacterial or fungal bodies (Figure 1c). Based on the clinical course and pathological findings, we diagnosed her as sirolimus-associated acneiform eruption. She was treated with topical 0.05% difluprednate ointment, doxycycline 100 mg/day, and fexofenadine 120 mg/day. The eruption mostly resolved after seven days of treatment, but it slightly continued during sirolimus treatment.

## Discussion

Clinical trials and cohort studies involving sirolimus and its derivative, everolimus, have consistently reported acneiform eruptions as a predominant adverse skin event. Most reported cases of sirolimus-induced acneiform eruptions are negative for bacterial cultures, and the few positive cases primarily involve skin resident commensals, suggesting these eruptions are typically sterile [5,6]. Notably, in a study of 80 kidney transplant recipients receiving sirolimus over an average duration of 18 months, 46% experienced acneiform eruptions [5]. Furthermore, a phase 1/2 randomized controlled trial investigating sirolimus in patients with lymphangioleiomyomatosis revealed a 1.58-fold increase in the incidence of skin adverse events, including acneiform eruptions, compared to controls [7]. Despite these observations, the dose dependence of these eruptions remains to be fully elucidated.

Both sirolimus and clarithromycin are metabolized by CYP3A [8]. Moreover, clarithromycin forms a MIC with CYP3A, which may result in a pharmacokinetic interaction that interferes with the clearance of sirolimus through CYP3A [9]. Patients who received clarithromycin during sirolimus treatment have been reported to exhibit increased blood levels of sirolimus [4]. Although there are no direct reports linking the concentration of sirolimus in the blood to the onset of acneiform eruptions, the occurrence of acneiform eruptions has been reported to be higher in groups receiving a high dose of sirolimus [5], suggesting that the increased blood levels of sirolimus due to clarithromycin administration might have led to the development of the acneiform eruption in this case. EGFR inhibitors are also known to cause folliculitis in up to 66.2% of treated patients [10]. Although the detailed mechanisms are not fully understood, it is anticipated that the strong inhibition of the EGFR/RTKs/PI3K/AKT/mTORC1 signaling pathways, which are involved in cell proliferation [11], is related to the development of folliculitis in both cases.

The limitations of this report include its basis on a single case study and the absence of monitoring sirolimus blood concentrations, and the recurrence of the acneiform eruption with the reinitiation of sirolimus alone complicates the identification of clarithromycin as the definitive trigger.

## Conclusions

In this case report, we present acneiform eruptions during treatment with sirolimus following the administration of clarithromycin, highlighting the potential involvement of drug-drug interaction between these two medications in triggering the eruption. Although further case accumulation is necessary, clinicians should be aware of the possibility of developing acneiform eruption during sirolimus treatment, especially when administered in conjunction with clarithromycin and other medications that inhibit CYP3A.

## References

[1] Sehgal SN (2003). Sirolimus: its discovery, biological properties, and mechanism of action. Transplant Proc.

[2] Campistol JM, de Fijter JW, Flechner SM, Langone A, Morelon E, Stockfleth E (2010). mTOR inhibitor-associated dermatologic and mucosal problems. Clin Transplant.

[3] Sturgill MG, Rapp RP (1992). Clarithromycin: review of a new macrolide antibiotic with improved microbiologic spectrum and favorable pharmacokinetic and adverse effect profiles. Ann Pharmacother.

[4] Kumondai M, Kikuchi M, Mizuguchi A (2023). Therapeutic drug monitoring of blood sirolimus and tacrolimus concentrations for polypharmacy management in a lymphangioleiomyomatosis patient taking two cytochrome P450 3A inhibitors. Tohoku J Exp Med.

[5] Mahé E, Morelon E, Lechaton S, Drappier JC, de Prost Y, Kreis H, Bodemer C (2006). Acne in recipients of renal transplantation treated with sirolimus: clinical, microbiologic, histologic, therapeutic, and pathogenic aspects. J Am Acad Dermatol.

[6] Kunzle N, Venetz JP, Pascual M, Panizzon RG, Laffitte E (2005). Sirolimus-induced acneiform eruption. Dermatology.

[7] Bissler JJ, Kingswood JC, Radzikowska E (2013). Everolimus for angiomyolipoma associated with tuberous sclerosis complex or sporadic lymphangioleiomyomatosis (EXIST- 2): a multicentre, randomised, double-blind, placebo-controlled trial. Lancet.

[8] Zanger UM, Schwab M (2013). Cytochrome P450 enzymes in drug metabolism: regulation of gene expression, enzyme activities, and impact of genetic variation. Pharmacol Ther.

[9] Mayhew BS, Jones DR, Hall SD (2000). An in vitro model for predicting in vivo inhibition of cytochrome P450 3A4 by metabolic intermediate complex formation. Drug Metab Dispos.

[10] Mok TS, Wu YL, Thongprasert S (2009). Gefitinib or carboplatin-paclitaxel in pulmonary adenocarcinoma. N Engl J Med.

[11] Wee P, Wang Z (2017). Epidermal growth factor receptor cell proliferation signaling pathways. Cancers (Basel).

